# Emulsifier-Free Acrylate-Based Emulsion Prepared by Reverse Iodine Transfer Polymerization

**DOI:** 10.3390/polym12030730

**Published:** 2020-03-24

**Authors:** Tao Huang, Qing-Xia Yuan, Shu-Ling Gong

**Affiliations:** College of Chemistry and Molecular Sciences, Wuhan University, Wuhan, Hubei 430072, China; huangtao2017@whu.edu.cn (T.H.); qingxiay@whu.edu.cn (Q.-X.Y.)

**Keywords:** self-emulsifying, acrylate-based emulsion, reverse iodine transfer polymerization, controlled radical polymerization, iterative one-pot method

## Abstract

The self-emulsifying acrylate-based emulsions with solid content 45 wt.% were prepared in 3.5 h by reverse iodine transfer polymerization (RITP), and the polymer molecular weight (*M*_n_) could be 30,000 g·mol^−1^. The influences of methacrylic acid (MAA) amount, soft/hard monomer mass ratio, and iodine amount on polymerization and latex were investigated. A moderate amount of ionized MAA was needed to stabilize the emulsion. Glass transition temperature (*T*_g_) was decreased with the increasing mass ratio of soft/hard monomer. A higher iodine amount resulted in lower *M*_n_. The increased *M*_n_ after chain extension of the polymer with water-insoluble monomers in iterative one-pot method proved the living of polymer. Compared with conventional emulsion polymerization, molecular weight (*M*_n_) could be controlled, and *M*_n_ of polymer synthesized in RITP emulsion polymerization is higher; emulsion of polyacrylate-containing hydroxyl monomer units prepared by RITP emulsifier-free radical polymerization is more stable. Good properties, such as hardness, water resistance, adhesion, and increased value of maximum tensile of films modified by reaction of polyacrylate with melamine–formaldehyde (MF) resin, indicated potential application in baking coating.

## 1. Introduction

Acrylate-based emulsions are widely used in industrial and consumer coating for the advantages of photo stability, resistance to hydrolysis, and excellent outdoor durability [1]. The conventional radical emulsion polymerization [2,3] is often used to prepared this kind of emulsion, and nonpolymeric emulsifiers, such as sodium dodecyl sulfate (SDS), are usually added into the system [4]. Dispersed system in emulsion is thermodynamically unstable, and the system can be stable when hydrophilic groups from emulsifiers are incorporated into the polymer [5]. In some emerging fields, emulsifiers such as nano emulsifiers [6,7,8,9,10] synthesized in emulsion can be used for preparing nanoparticles with special function. With the use of nonpolymeric emulsifier, acrylate-based latex with high solid content (over 40 wt.%) and low viscosity can be prepared. In emulsion polymerization, water-insoluble monomer can take part in the polymerization with the emulsification of emulsifier that stabilizes the latex particle, and the synthesized polymers can be dispersed in water. But when the emulsions are used as metal coatings, the migration of the residual emulsifier migration to the surface of the polymer films may result in shrinkage, a decrease of the coating gloss, etc., and metal surface can confer water sensitivity to the films, leading to loss of the protective effect of coating [11]. Polymeric emulsifier, which can be chemically bonded to the polymer chain, is then introduced into the emulsion system [12,13,14,15]. For emulsion prepared by conventional radical polymerization [12], polymeric emulsifier SE-10 takes part in the stabilization of the polyacrylate emulsion. However, the sulfonic acid group from SE-10 can react with metal, and the sulfonic acid group may decrease water resistance of the modified film. In addition, the molecule weight in this kind of emulsion was no more than 11,000 g·mol^−1^, and the relatively low molecular weight may restrict further improvement of mechanical property of the modified film. The surfactant-free emulsion of acrylate-based polymer with high molecular weight can be prepared by conventional radical polymerization, using of 5–10 wt.% acrylic acid (AA), but the limitation is the solid content is only 34.2 wt.%, lower than 40 wt.% [16]. Therefore, exploring synthesis methods to synthesis polyacrylate emulsion with high solid content and high molecular weight is necessary.

Conventional radical polymerization and controlled radical polymerization (CRP) belong to radical polymerization. The controllability lies in the stage that mediates the rates of propagation and termination in polymerization. There existed some substance that can be reversibly bonded to the growing radical of the polymer chain for CRP, while no analogous functional substance existed for conventional radical polymerization [4]. From the point of reaction mechanism, the radical density in conventional radical polymerization is higher than that of CRP for having no substance that can combine with the radical to form dissociable covalent dormant seed, so conventional radical polymerization loses control in molecular weight [17]. In the emulsion system, the acrylate-based polymers can be synthesized by CRP, including nitroxide-mediated polymerization (NEM) [18,19,20,21], reversible addition-fragmentation chain transfer (RAFT) polymerization [22,23,24], atom-transfer radical polymerization (ATRP) [25,26,27,28], and reverse iodine transfer polymerization (RITP) [29,30,31,32,33,34]. In CRP emulsion polymerization such as NEM, RAFT, and ATRP, well-defined polymers with controlled molecular weight, composition, chain architecture, and site-specific functionality can be prepared [17]. Both ATRP and RAFT generally owe faster polymerization kinetics and lower polymerization temperatures than that of NEM, and a broader range of monomers can be polymerized [35]. In the NEM polymerization of methacrylate monomers, the addition of a small amount of a comonomer with a lower activation deactivation equilibrium constant (K) is needed to decrease the overall concentration of propagating radicals for high concentration of propagating radicals can cause irreversible termination reactions [36,37,38,39,40]. Emulsion prepared by RAFT polymerization is sensitive to the pH of the environment for the structure of thioester in the end of chain, and the synthesized chain transfer agent is needed before the polymerization. ATRP requires transition metal compound and ligand that forms a complex with the transition metal, to modify catalyst solubility, stability, and activity [41]. RITP does not require complicated chemicals [32] and is very cheap compared to other living radical polymerizations, such as NEM, ATRP, and RAFT [29]. Compared to ATRP and RAFT, the transfer agents are synthesized in situ in the process of RITP polymerization [29], and common initiator can be used. In brief, RITP does not require the synthesis nor storage of control agents before polymerization [29]. For emulsion prepared by RITP polymerization with nonpolymeric emulsifier SDS, the measured *M*_n_ can be over 20,000 g·mol^−1^ in 7.7 h, with 90 wt.% monomer conversion, and the introduction of iodine leads to lower molecular weight distribution, compared to conventional emulsion polymerization [29], showing that RITP could be an effective way to prepare emulsion with relatively high molecular weight and high monomer conversion [29,32].

**Figure 1:**
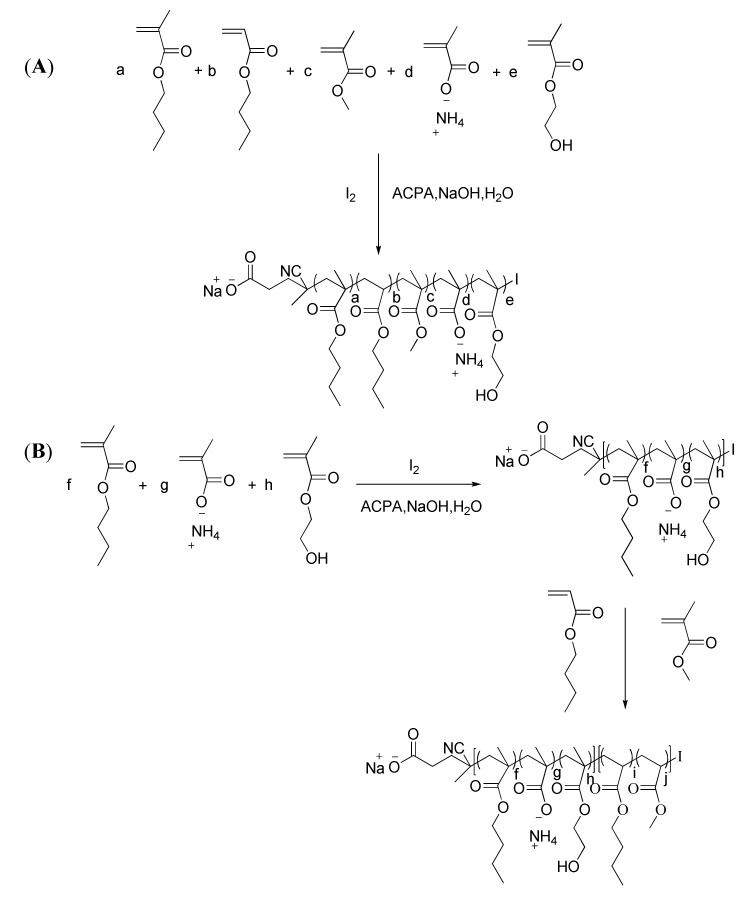
(**A**) Synthetic route of copolymer in reverse iodine transfer polymerization (RITP) and (**B**) chain extension in iterative one-pot emulsion copolymerization.

In CRP, polymer with methacrylic acid units in the main chain could be used as emulsifier and chain transfer agent to synthesis acrylate-based block copolymer in emulsion system, and the latex was stable [42,43]. Furthermore, the chain transfer agent can be synthesized in situ for RITP. In this research study, high solid content emulsion prepared by copolymerization of acrylate-based monomers with ammonium salt of methacrylic acid (MAA) in one pot by RITP Polymerization (Figure 1) was studied by changing the amount of MAA, soft monomer/hard monomer mass ratio, and amount of iodine. In addition, chain extension reaction was conducted by addition of monomer mixture into the latex in Iterative one-pot method. The emulsion was characterized by viscosity, dynamic light scattering (DLS), gel permeation chromatography (GPC), thermo gravimetric analysis (TG), and differential scanning calorimetric (DSC). As shown in Figure 1, there was no nonpolymeric emulsifier added to either the random copolymerization or chain extension. The stable white emulsions indicate self-emulsifying ammonium salt of MAA can stabilize the latex. The experimental *T*_g_ of drying latex could be tuned by changing the mass ratio of soft/hard monomer according to the Fox equation [44], and *T*_g_ ranged from 6.1 to 43.9 °C. The *M*_n_ of polymer could be tuned by changing the amount of iodine. The chain extension reaction proved the living of the polymer. The application of emulsion was conducted by the reaction of the polyacrylate with MF resin to prepare modified film. This work may provide potential direction to application of emulsion in coating used for metal surface protection.

## 2. Materials and Methods

### 2.1. Materials

Methyl methacrylate (MMA), methacrylic acid (MAA), n-butyl acrylate (BA), n-butyl methacrylate (BMA), ammonia solution (25–28 wt.%), iodine, sodium hydroxide, and p-toluenesulfonicacid (TsOH) were purchased from Sinopharm Chemical Reagent Co., Ltd. (Shanghai, China), analytical regent (AR). Methacrylicacid-β-hydroxyethyl ester (HEMA) was purchased from Tianjin Institute of Chemical Reagents (Tianjin, China), AR. N,N-dimethylethanolamine (DMEA) was purchased from Tianjin Kemiou Chemical Reagent Co., Ltd. (Tianjin, China), AR. The initiator 4,4′-Azobis (4-cyanovaleric acid) (ACPA,98%, AR) was purchased from Energy Chemical and contains ca. 20% water. Hexamethylolmethymelamine (HMMM) was provided by H. J. Unkel Co., Ltd. (Zhuhai, China). The monomers MMA, BA, and BMA were purified by washing with 10 wt.% aqueous sodium hydroxide solution 4 times, and then by the deionized water 4 times. Other materials were used as received.

### 2.2. Methods

#### 2.2.1. Emulsifier-Free Copolymerization of Acrylate

In a typical example illustrated in Figure 1A, 2.618 g (22.55 mmol) of HEMA was added or not, 6.162 g (43.33 mmol) of BMA, 2.054 g (20.52 mmol) of MMA, 1.027 g (8.01 mmol) of BA, 12.121 g of deionized water, and 273 mg (1.074 mmol) of I_2_ were added into a 100 mL flat-bottom flask and then stirred by a magnetic stirrer for 15 min, in order to dissolve I_2_ in organic phase. Thereafter, 1.664 g (19.33 mmol) of MAA, 4.042 g H_2_O, and 1.39 g ammonia solution (20.43–22.84 mmol NH_3_) were added into the flask, and the mixtures were stirred by a magnetic stirrer for 15 min. After that, 3.461 g (1.235 mmol) of ACPA 10 wt.% solution (2.25 g ACPA, 642 mg NaOH, and 19.608 g H_2_O) was added. The total monomer mass content by weight in theory is 40 wt.%. The reaction system was deoxygenated by bubbling with high-purity nitrogen for 25 min in room temperature. With the atmosphere of high purity nitrogen provided by balloon, the solution was heated to 80 °C and maintained at this temperature for 210 min, under stirring. The reaction was ceased by exposure to air. The monomer conversion and solid content was determined by gravimetric analysis.

When the mass ratio of the monomer or the mass of the I_2_ changed, the procedure was as the above.

#### 2.2.2. Iterative One-Pot Emulsion Copolymerization for Chain Extension

Firstly, 4.451 g (31.30 mmol) of BMA, 9.027 g of deionized water, and 409 mg (1.611 mmol) I_2_ were added into a 100 mL flat-bottom flask and then stirred by a magnetic stirrer for 15 min. Thereafter, 1.664 g (19.33 mmol) of MAA, 4.233 g H_2_O, and 1.39 g (20.43–22.84 mmol NH_3_) ammonia solution were added into the flask and stirred for 15 min. After that, 5.192 g (1.852 mmol) of ACPA 10 wt.% solution was added. The reaction system was deoxygenated by bubbling with high-purity nitrogen for 25 min, at room temperature, with stirring. With the atmosphere of high-purity nitrogen provided by balloon, the solution was heated to 80 °C and maintained at this temperature in the reaction time, with stirring. Solution sample was taken when time reached 140 min (the first stage) and exposed to the air. Thereafter, the nitrogen gas–saturated mixture solution comprising 2.739 g (21.37 mmol) BA and 2.054 g (20.52 mmol) of MMA was added into the flask and stirred for 90 min (the second stage).

When HEMA was added into the system, the procedures were as the above. The synthesis of copolymer containing HEMA in the chain is shown in Figure 1B. The reaction time in the first stage was 170 min, and that in the second stage was 90 min.

#### 2.2.3. Modification of the Emulsion by MF Resin

Firstly, 2.5 g of emulsion, 0.96 g of deionized water, and 0.64 g of HMMM were added into 25 mL round flask, with magnet stirring, at room temperature. Then, 85 μL of TsOH solution (10 wt.%) was added dropwise. The mixture was stirred for 1 h. After that, the mixture was coated on clean tinplate and coverslip, with water evaporating for 2 h. Thereafter, the tinplate and coverslip were heated at 80 °C for 120 min and then 150 °C for 40 min. The modified film was prepared by the above procedure.

### 2.3. Characterizations

#### 2.3.1. Viscosity

The Viscosity of emulsion was tested by DV-79 digital viscometer (Shanghai Ni Run Intelligent Technology Co., Ltd, Shanghai, China) with E-type rotor or F-type rotor at 25 °C, when the rotational rate of the rotor was 75 or 750 rpm.

#### 2.3.2. Particle Size

The sample of the emulsion was diluted to 1000 times by volume, with adding deionized water. The particle size and polydispersity characterizing the particle size distribution of the diluted sample were measured by a Zetasizer Nano ZS laser particle sizer, Malvern Instruments Ltd. (Shanghai, China) with a 90 degree scattering angle, at 25 °C.

#### 2.3.3. Gel Permeation Chromatography

After the further neutralization of the emulsion by DMEA, the excess DMEA was removed by rotary evaporation at 60 °C, under reduced pressure, and then the dried polymer was dissolved in THF (20 mg/mL), and the solution was filtrated by organic phase filter head. The number average molecular weight (*M*_n_), the weight average molecular weight (*M*_w_), and the index of molecular weight distribution (*Ɖ* = *M*_w_/*M*_n_) of polymer were determined by Waters-515 gel permeation chromatograph from the US WAERS Corporation (Milford, MA, USA), with THF as eluent, at a flow rate of 1.0 mL·min^−1^ at 25 °C, and calibrated by Polysilane standards.

#### 2.3.4. Monomer Conversion

The determination of monomer conversion was as follows:

(1) $$\mathrm{Conversion}\%=\frac{m\left(\mathrm{Dried}\;\mathrm{emulsion}\right)}{m\left(\mathrm{Emulsion}\;\mathrm{solution}\right)\times w\left(\mathrm{Total}\;\mathrm{monomer}\right)}$$

where *w* (Total monomer) is the total mass content of monomer in weight.

#### 2.3.5. Thermogravimetric Analysis

The sample was prepared by drying the emulsion under the radiation of an infrared lamp for 24 h, and the thermal stability of the sample was measured by TG 209 F1 thermogravimetric analyzer from NETZSCH, Selb, Germany, under nitrogen atmosphere. The operation temperature ranged from 30 to 800 °C, and the heating rate was 10 °C min^−1^. The corresponding temperature *T*_5%_ that 5% weight loss of the drying sample of the emulsion was measured, and so were *T*_10%_ and *T*_50%_. *T*_max_ is the maximum thermal decomposition rate temperature.

#### 2.3.6. Differential Scanning Calorimetry

The glass-transition temperature (*T*_g_) was measured by TA Q20 DSC Instrument (TA Instruments, New Castle, DE, USA) under nitrogen atmosphere, and the measuring temperature range was from −15 to 120 °C, while the heating and cooling rate was 10 °C·min^−1^.

#### 2.3.7. Fourier Transform Infrared Spectroscopy

The sample was prepared in the way as is mentioned above, in the section of GPC Sample. The infrared spectrum of the sample film was measured by Thermo Nicolet Fourier transform infrared spectroscopy (FTIR) (Thermo Fisher, Waltham, MA, USA), using Attenuated Total Reflectance (ATR) method in Smiths Detection, equipped with diamond. The scanning range was 4000–500 cm^−1^.

#### 2.3.8. Hardness of the Modified Film

The measurement of hardness of the film by pencil test was based on the procedure directed by China National Standard GB/T 6739-2006/ISO 15184:1998.

#### 2.3.9. Scratch Experiment of the Modified Film

The adhesive property is shown by the resistance that the film is detached from the substrate; test rank is based on the procedure directed by China National Standard GB/T 9286-1998 equivalent ISO 2409:1992.

#### 2.3.10. Water Resistance of the Modified Film

The resistance of the film to water was tested via a method based on the procedure directed by China National Standard GB/T 1733-1993.

#### 2.3.11. Tensile Property

Tensile property of dried polyacrylate film or modified film was tested by Instron 5967 Universal Tensile-Compressive Tester, at 10% length per minute, or by Electronic Universal Testing Machine from MTS SYSTEMS (China) Co., Ltd (Shanghai, China) with SANS-Power Test software, at 10 mm·min^−1^.

## 3. Results and Discussion

### 3.1. Effect of HEMA on Emulsion Prepared by Random Copolymerization of Acrylate

The hydroxyl group from the HEMA unit in polymer chain can react with MF resin to improve the mechanical property of the polymer. HEMA homopolymer is partially soluble in water at pH 6.5, in the whole temperature range from 0 to 80 °C, when degree of polymerization (DP_n_) is less than 20, but it is not soluble in water in this temperature range when DP_n_ is higher than 20 [45]. When water-insoluble monomer is polymerized with HEMA, the solubility of the copolymer may not be analogous to that of HEMA homopolymer. Therefore, emulsifier or polymeric monomer must be needed to stabilize the emulsion of polyacrylate with HEMA copolymerized. In the previous work of our group, the mass ratio of HEMA in overall monomer of the recipe with best combination property was 20 wt.%, and ammonium persulphate (APS) was chosen as initiator, with polymeric monomer SE-10 used for emulsifier [12]. In this paper, the exploratory experiment of RITP polymerization that APS was added into the emulsion system with monomer styrene (St), HEMA, BA, and polymeric emulsifier SE-10 was done, but the latex was unstable; the color of the latex was yellow. The excess APS could oxidize iodide produced by the hydrolysis of iodine in water [33]; the color of the emulsion prepared by the exploratory experiment was not white. Choosing an appropriate initiator for RITP emulsion is necessary. Initiator ACPA cannot oxidize iodide in RITP polymerization of BA, and the emulsion is stable and white [32], so ACPA was chosen as the initiator in the polymerization of this study.

Without nonpolymeric emulsifier, AA is preferentially consumed because of its higher reactivity in the early stages of the RITP polymerization, and the amphiphilic gradient copolymers can be formed in situ, and the polymer latex can be stabilized in the emulsion system with initiator potassium persulfate (KPS) [34]. The carboxyl group from AA units or MAA units of polymer chain can react with MF resin [44]. Based on this cognition, MAA may be introduced into the emulsion-based RITP polymerization, to use as the polymeric emulsifier.

In Table 1, because only 0.204 g of ammonia solution (2.99 mmol NH_3_) was added for the polymerization without HEMA, a lot of coagulation existed in the end of the reaction time when most of MAA (19.33 mmol) in emulsion was not ionized. While MAA solution is adjusted to pH 8–9, the emulsion from comparing experiment in the mass ratio of 7/2 in Table 2 was stable in the reaction time and in six months, indicating that MAA must be ionized to stabilize the emulsion. With the increasing of neutralization degree that can increases the electrostatic repulsion of the dissociated MAA, the polymerization rate of MAA is found to decrease [46]. Furthermore, the excess ammonia solution may react with iodine, decreasing the participation of I_2_ in the regulation of polymerization; therefore, the amount of ammonia solution should be controlled in order to adjust the emulsion to neutral pH.

For emulsion with HEMA, the hydrogen bonding between the hydroxyl from HEMA units in the polymer and the carboxyl from AA units in the polymer results in the formation of interpolymer complexes [47], which reduces the solubility of the emulsion. Thus, the solution of MAA solution should be adjusted to neutral pH in order to restrain the formation of interpolymer complexes. As shown in Table 1, when the mass ratio of BMA/MMA was 1/3 or 2/1, a little coagulation existed in the emulsion, although heating temperature was decreased to 75 °C 60 min after. When the mass ratio was 3/1 or 4/1, the emulsion was stable, and the emulsion could flow fluently. When the mass ratio was 7/2, the emulsion could not flow fluently with the additional 30 min reaction time at 75 °C; meanwhile, a decrease of the mass of BMA and introduction of BA into the emulsion and keeping the value of [*m*(BMA)+*m*(BA)/*m*(MMA)] to 7/2 could make the emulsion stable. The result above indicates that, without a soft monomer such as BA, polymeric monomer such as ammonium salt of MAA has limitations in stabilization of high monomer mass content (over 40 wt.%) emulsion when hard monomer participates in emulsion polymerization.

For the series shown in Table 2, the mass ratio of BA/MMA stayed at 1/2, and the conversion was more than 99% without flocculation, while the solid content was higher than 39 wt.%. The latex was white in the end of the reaction, indicating that I_2_ had been completely consumed. In the stage of polymerization with HEMA or without HEMA in Table 2, there existed no obvious flocculation that could deteriorate the stability of emulsion.

The target theory molecular weight (*M*_n,th_) is calculated by the equation attached to Table 2. According to RITP mechanism [30,48], one molecule of iodine will control two polymers for terminal group of iodine atom, and initiator group is chemically attached to the beginning of the polymer chain. Without the addition of HEMA, the experimental molecular weight (38200 g·mol^−1^) of the latex was much higher than the target theory molecular weight (6140 g·mol^−1^) when the total mass ratio of BMA/MMA and BA/MMA was 3/1. This phenomenon also existed in the total mass ratio of 7/2 or 4/1 without HEMA and three ratios with HEMA. The reason of this deviation is partly the hydrolysis of iodine in water which decreases the amount of iodine reacted with the propagation radical [30,31,32,33,34,49,50]. In addition, the ammonium salt of MAA and that in the polymer skeleton could be decomposed at 80 °C, leading to the generating of ammonium hydroxide, which can react with HI produced by the hydrolytic disproportionation of I_2_. In a comparison experiment conducted without the addition of ammonium hydroxide in the ratio of 3/1(0 g HEMA, Table 2), the molecular weight (*M*_n,GPC_) was 9290 g·mol^−1^, *Ɖ* 3.03, and monomer conversion was 87.3% in 160 min, when the emulsion was just unstable, with magnetic agitator stirring un-smoothly; with polymerization time on, the emulsion was not stable, although MAA (12.30 wt.% in total monomers) were used, indicating that ionizable chain-ends from the initiator have limitations in the electrostatic stabilization of the latex in the studied polymerization system. Without ammonium hydroxide, the deviation of experimental *M*_n_ (9290 g·mol^−1^) from the theoretical *M*_n_ (5390 g·mol^−1^) is much lower than that in the ratio of 3/1 in (*M*_n,GPC_ = 38,200 g·mol^−1^), but the former *Ɖ* (3.03) is larger than the later (*Ɖ* 1.48). The result of the comparison experiment indicates that the addition of ammonium hydroxide was the reason for the deviation of *M*_n_ in RITP polymerization by emulsion of this paper. Based on the abovementioned deviation of molecular weight, the designed *M*_n_ ranges from 4000 to 7000 g·mol^−1^, in order to synthesis the polymer with experimental *M*_n_ ranging from 20,000 to 50,000 g·mol^−1^.

For the polymerization in Table 2, *Ɖ* is less than 2; this result shows narrow molecular distribution. Without the addition of ammonium hydroxide in the emulsion system when HEMA took part in the polymerization, the emulsion was unstable because of the formation of interpolymer complexes derived by the hydrogen bonding between the hydroxyl of the polymer and the carboxyl of the polymer in acid condition [47,51]. With the addition of HEMA and ammonium hydroxide in Table 2, the emulsion was stable in the reaction time, and the latex was white. Comparing the results of three ratios in Table 2, the value of experimental *M*_n_ is smaller than the one with no addition of HEMA, but the value of *Ɖ* is larger. As illustrated in the total mass ratios of 3/1 and 4/1, in Table 2, the number mean diameter (*d*_p_) of emulsion with HEMA is larger than that without HEMA because the hydrophilic HEMA units thickening the hydration layer on the emulsion particle surface [52]. The emulsion with HEMA owes higher viscosity than that without. The reason is for this is that the hydrogen bonds between HEMA units and water, as well as that between the hydroxyl groups of the HEMA units and the HEMA units [52].

In Table 3, the corresponding *T*_5%_ and *T*_50%_ in the total mass ratios of 7/2 and 4/1 were increased when HEMA was taken part in the emulsion polymerization, indicating that the thermal stability emulsion polymer of certain mass ratio with the addition of HEMA is better than that without HEMA. The experimental *T*_g_ in the three mass ratios was in the range from 28.0 to 36.3 °C. The *T*_g_ in the total mass ratio of 3/1 was higher than that of 7/2, but lower than that of 4/1. The glass transition temperature (*T*_g_) of random copolymer can be calculated via the Fox equation [44], and more hard monomers lead to higher *T*_g_. This tendency is only obvious from the total mass ratio of 7/2 to that of 3/1, but opposite from the ratio of 4/1 to that of 7/2.

As show in Table 4, after modification, the pencil hardness rank of the cured film could reach 3H, and adhesive rank of the film could reach 0. Before modification, the drying latex of the random copolymer could be dispersed in water at room temperature, and the solution was turbid. By contrast, the film of the modified copolymer was translucent in boiled water, and the film was not dissolved in water for the crosslinking structure of the polymer, indicating that water resistance of the film is good.

*M*_n,GPC_, *d*_p_, and the viscosity of the emulsion without HEMA in Table 2 is prominent different from those with HEMA. The viscosity in the total mass ratio of 7/2 (see Table 2) is in the middle range, but the *d*_p_ is smaller than that without HEMA. In conclusion to this part, it appears that the good results combining *M*_n,GPC_, *Ɖ*, *d*_p_, viscosity, pencil hardness, adhesive property, water resistance, and *T*_g_, with *T*_5%_ are obtained in the total mass ratio of 7/2 (see Table 2). Therefore, the recipe in the total mass ratio of 7/2 (see Table 2) was chosen for further research in the following section of this article.

### 3.2. The Influence of the Amount of MAA on Polyacrylate Emulsion

At neutral pH, the acrylic acid would be in the ionic form (p*K*_a_ = 4.25), and then the stabilization of the polymer latex is better [34]. In the previous work of our group, ammonia solution was added to the pre-emulsion, to ionize MAA, and in the polymerization period, to neutralize hydrogen ion derived by the decomposition of initiator APS [12,15]. Therefore, in order to ionize MAA to prepare emulsion by RITP Polymerization, ammonia solution was added into the MAA solution, to neutral pH.

As illustrated in Table 5, the emulsion was not stable in 0.5 *m*(MAA)_0_, indicating that the emulsifying effect of neutralized ACPA is limited and more self-emulsifying monomer is needed to ensure the stability of the reaction system. With the addition of more MAA, as shown in Table 5, the conversion was more than 99%, and the emulsions were stable in the reaction time and six months after the reaction time, manifesting the emulsification of ammonium salt of MAA. *Ɖ* was 1.53 when *M*_n,GPC_ was 32,700 g·mol^−1^ with high viscosity, showing the feasibility of RITP in high monomer content (40.4 wt.%) emulsion polymerization. The particle size of the latex did not decrease with the increasing of MAA, and *d*_p_ ranged from 357 to 501 nm. In 1.0 *m*(MAA)_0_, as illustrated in Table 5, the viscosity was the least, and the emulsion flowed fluently. When the additional amount of MAA deviated from the reference amount, the viscosity was high and the emulsion flowed un-fluently.

As shown in Table 6, *T*_50%_ and *T*_max_ remained nearly unchanged in the five mass ratios. *T*_5%_ of sample from 1.1 *m*(MAA)_0_ (141 °C) was the lowest, while *T*_5%_ of the sample with 0.9 equivalent of the reference MAA (205 °C) was the highest. From the prospective of thermal stability, the sample with 0.9 *m*(MAA)_0_ was the best.

As shown in Table 7, the pencil hardness rank of the film was kept at 2H with the increasing amount of MAA. However, the adhesive property was degraded when the amount of MAA was over the reference amount (1.0 *m*(MAA)_0_) (see Table 7). Thereafter, the color of cured film became white, in and out of the boiled water, indicating that the water resistance became worse when the maximum amount of MAA (1.2 *m*(MAA)_0_) was used in the polymerization. These results suggest that the excess amount ammonium salt of MAA used in the polymerization can increase the hydrophilic property of polymer, leading to the decreasing of water resistance of the film. Superfluous MAA led to the difficulty of stirring of the emulsion, which may hinder the dispersing of the addition of monomer mixtures in the reaction time for chain extension.

In conclusion to this part, a moderate amount of ionized MAA is needed to stabilize the emulsion. It appears that the best results combining *M*_n_,_GPC_, *Ɖ*, *d*_p_, low viscosity, emulsion appearance, pencil hardness, adhesive property, and water resistance with moderate thermal stability are obtained when one equivalent of the reference MAA is added into the emulsion polymerization system.

### 3.3. The influence of Soft/Hard Monomer Mass Ratio on Polyacrylate Emulsion

For random free radical copolymerization, the *T*_g_ can be estimated by the Fox equation [44]. As is well-known, the *T*_g_ of the polymer can determine the application temperature scope. The monomer with a high *T*_g_ of homopolymer refers to hard monomer, while a low *T*_g_ refers to soft monomer. The *T*_g_ of the homopolymer of BA is –54 °C, BMA 20 °C, HEMA 55 °C, MMA 105 °C, and MAA 185 °C [44]. BMA and BA belong to soft monomer, while MMA belongs to hard monomer. MAA has adhesive property, and HEMA shows hydroxyl, which can react with amino MF resin to prepare film with a crosslinking structure. When the total amounts of components are kept the same, the mass ratio of the soft/hard monomer could be adjusted by changing the amount of BA, BMA, and MMA, and then the recipes of copolymer with a varied *T*_g_ could be designed.

**Figure 2:**
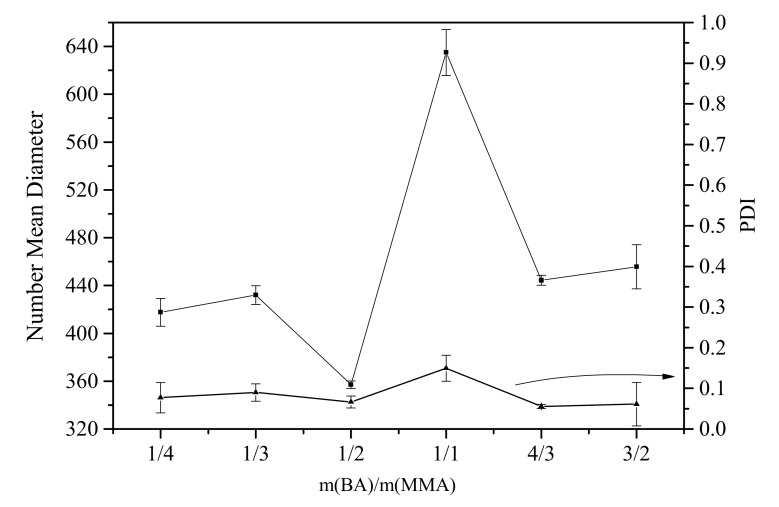
Particle size and polydispersity index (PDI) of particle with different soft/hard monomer ratio.

As shown in Table 8, the monomer conversion is more than 97%. The value of *M*_n,GPC_ was around 27,000 g·mol^−1^. However, when the mass ratio of soft/hard monomer was 1/2, the molecular weight distribution (1.62) was the narrowest. The viscosity was more than 1500 mPa.s in the ratios of 1/4, 1/3, and 1/1. From the error bars shown in Figure 2, the measurement of every mass ratio was conducted three times. The diameter was around 420 ± 60 nm, except for the particle in the ratio of 1/1. The size in the ratio of 1/1 is larger than that in other five ratios. Changing soft/hard monomer ratio may not make obvious difference in size. However, there is also work reported that the particle size was not affected by mass ratio of BA/MMA [53,54,55] in conventional emulsion-free radical polymerization. In Figure 2, we also found PDI was around 0.06 ± 0.03, except for the particle in the ratio of 1/1. PDI in the ratio of the 1/1 is larger than that in other five ratios. In most case of this section, changing soft/hard monomer ratio may not make obvious difference in size and PDI. The reason is still unknown.

As shown in Table 9, *T*_50%_ and *T*_max_ remained almost unchanged in the six mass ratios. *T*_5%_ of the sample with the ratio of 1/1 was the lowest, while *T*_5%_ of the sample with the ratio of 3/2 was the highest. From the prospective of thermal stability, the ratio of 3/2 was the best. As the mass ratio of the soft/hard monomer was increased from 1/4 to 3/2, the experimental *T*_g_ decreased from 43.9 to 6.1 °C. These *T*_g_ results accord with the tendency from the Fox equation, that a greater amount of soft monomer leads to higher *T*_g_ of random copolymer.

In Table 10, there existed good hardness and adhesive properties in the ratio between 1/3 and 1/1. When the ratio was 1/4, as shown in Table 10, the hardness of the film (3H) was of the highest rank. As mentioned above, MMA belongs to hard monomer, and PMMA is stiff at room temperature, so the modified random polymer in this paper with a high amount of MMA units in its polymer chain may show its hardness at room temperature. Before putting the drying film into boiling water, the film was translucent and pale yellow. After putting the film into the boiling water, the film from the ratio of 1/2 and 1/1 was translucent, proving the film possesses good water resistance.

In conclusion to this part, it appears that the best results in terms of *M*_n,GPC_, *Ɖ*, *d*_p_, low viscosity, pencil hardness, adhesive property, water resistance, thermal stability, and glass transition temperature are obtained when the mass ratio BA/MMA is 1/2.

### 3.4. The influence of Iodine on Copolymerization of Acrylate

In emulsion polymerization of BA in water, increasing the initiator may increase the polymerization rate and level of branching due to intermolecular transfer to polymer [56,57]. Therefore, the mole ratio of [ACPA]/[I_2_] should not be too high in the polymerization.

As shown in Table 11, the experimental molecular weight was higher than that of the theory. The experimental molecular weight was decreased with the increase of I_2_, and this result is in accordance with the rule that there is an inverse proportional relationship between the molecular weight of polymer and the amount of I_2_ in RITP Polymerization [32,48]. The latex was white and stable in the reaction time with no HEMA copolymerized, and the solid content was higher than 36 wt.%. Except for *d*_p_ in 1.1 *m*(I_2_)_0_, *d*_p_ ranged from 381 to 400 nm. When *m*(I_2_)/*m*(I_2_)_0_ was 1.1, the monomer conversion was slightly lower, but the diameter of particle was the least, and the PDI value was in the middle range. The above result shows that self-emulsified ionized MAA could be benefit from the stabilization of the latex and the molecular weight can be tuned by the amount of iodine in RITP.

### 3.5. Iterative One-Pot Emulsion Copolymerization

As illustrated in Figure 1B, MAA was ionized by neutralization with ammonia solution. As shown in Table 12, random copolymer was synthesized with conversion over 99%. The color of the latex of Poly (MAA-co-BMA) or Poly (MAA-co-HEMA-co-BMA) was white, proving that I_2_ was consumed in the first stage. Poly (MAA-co-BMA) or Poly (MAA-co-HEMA-co-BMA) is amphiphilic from the point of primary chemical structure. When the reaction time was up, the latex was stable, showing that Poly (MAA-co-BMA) or Poly(MAA-co-HEMA-co-BMA) could play the part of emulsifier and stabilizer in the emulsion polymerization. From the propagation reactivity prospective of the monomer added into the second stage, the chain extension did not conduct successfully for the disturbance of residual oxygen in the mixture solutions while there was water in the mixture solution. Moreover, when the mixture solution consisted of BMA and MMA, the latex was not stable and the layer was separated, as it was in the system of the mixtures of HEMA and water. It is well-known that the mixture solution containing volatile monomer cannot be purged by bubbling with high-purity nitrogen for long time, and a 30 min purging time is suggested. Thus, the deoxygenated mixture solution of BA and MMA was added into the emulsion, and iterative one-pot synthesis of block copolymer was conducted in the second stage.

In Table 12, the conversion was over 99%, and the index of molecular weight distribution was narrowed after chain extension; however, the *d*_p_ and PDI was increased. After chain extension, the solid content of the emulsion was higher than 40 wt.%. The theory molecular weight distribution in controlled living radical polymerization approach to 1. RITP Polymerization belongs to controlled living radical polymerization [29]. The molecular weight distribution of block copolymer in RITP emulsion polymerization is generally high (1.5–1.9) [31,32], but lower than that of the random copolymer without iodine. Therefore, the dispersity is higher than that expected for a controlled living radical polymerization. When HEMA was not added into the first stage, the reaction time should have been shortened. In the second stage, the *d*_p_ of the stable emulsion increased from 283 to 323 nm, or 243 to 414 nm, indicating polymer particles had grown by chain extension. Meanwhile, the increased *M*_n_ and decreased *Đ* manifest that the emulsion polymer in the first stage can be used as macromolecular chain transfer agent to control the copolymerization of water insoluble monomer BA and MMA. The emulsion of block copolymer is stable in the reaction period, indicating that polymer prepared by RITP Polymerization can act as macro-emulsifier when polymeric emulsifier takes part in emulsion polymerization. In conclusion, these results prove the living of polymer chains of Poly(MAA-co-HEMA-co-BMA) or Poly(MAA-co-BMA) when MAA was ionized by neutralization with ammonia solution.

From Table 13, it is apparent that *T*_5%_ and *T*_10%_ of block copolymer were higher than that of the polymer in the first stage. However, this is the contrary in the case for the *T*_50%._ From the viewpoint of TGA measurement, the thermal stability of the block copolymer was better than the seeded polymer in the first stage.

The rank of pencil hardness for cured film from modified block copolymer containing HEMA was 2H, and the rank of adhesive property was 1. The cured film immersed in boiling water was translucent, indicating good water resistance of the modified film.

In conclusion of this part, the living of the seeded polymer was proved by the chain extension reaction in iterative one-pot method. In addition, the copolymer after chain extension reaction showed larger molecule weight, lower molecular weight distribution, larger diameter, and better thermal stability than the seeded polymer. Moreover, the copolymer after chain extension reaction with HEMA had the property of high rank of hardness, high rank of adhesive property, and good water resistance.

### 3.6. Infrared Spectra of the Copolymer

**Figure 3:**
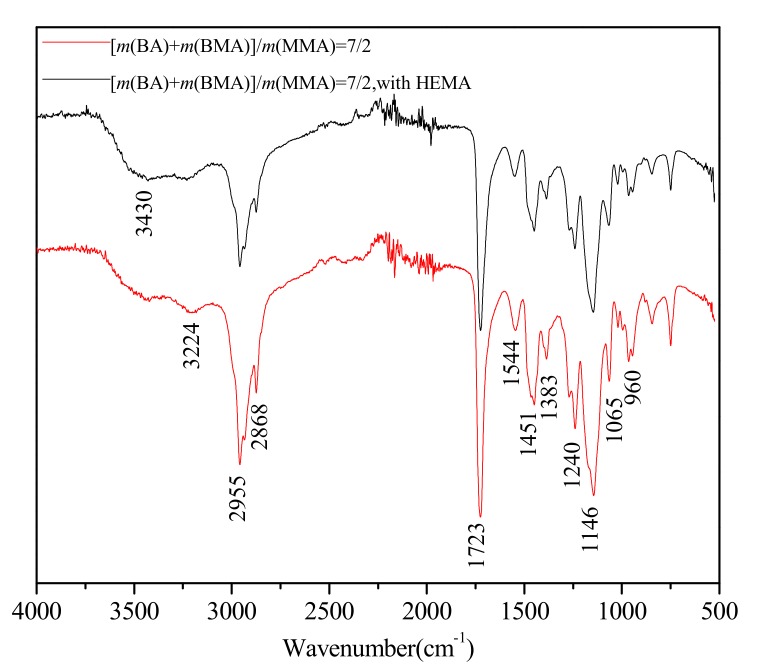
Fourier transform infrared spectroscopy (FTIR) spectra of copolymer without methacrylicacid-β-hydroxyethyl ester (HEMA) and copolymer with HEMA.

The FTIR Spectra of Polyacrylate are shown in Figure 3. The broad absorption peak, at 3430 cm^−^^1^, is ascribed to O-H stretching vibration. The hydroxyl exists in HEMA units and the tertiary ammonium salt of MAA units. N,N-dimethylethanolamine(DMEA) was introduced to partly neutralize MAA, so additional hydroxyl existed in the tertiary ammonium salt of MAA units. The broad absorption peak, 3224 cm^−1^, is ascribed to N-H stretching vibration of NH_4_^+^ and NH_4_^+^ derives from ammonium salt of MAA units in polymer chain. Whether HEMA was added or not, the peak at 3430 and 3224 cm^−1^ did not change peak shape in an obvious way. The two peaks at 2955 and 2868 cm^−1^ are caused by the C-H stretching vibrations of –CH_3_ and –CH_2_ groups, respectively. In addition, two peaks at 1451 and 1383 cm^−1^ are ascribed to the C–H bending vibrations of –CH_3_ and –CH_2_ groups, respectively. The strong absorption peak at 1723 cm^−1^ is caused by the stretching vibration of the carbonyl ester C=O. The peak at 1544 cm^−1^ is ascribed to the asymmetric stretching vibration of carbonyl anion COO^−^. Asymmetric stretching vibration and symmetric stretching vibration of the ester group C–O–C occurs in the bands of 1240 and 1065 cm^−1^, respectively. The strong absorption peak at 1146 cm^−1^ is caused by the bending vibration of C–O–H. The weak absorption peak at 960 cm^−1^ is ascribed to –CH_3_ rocking vibration.

### 3.7. Tensile Property of Polymer Emulsion Film

**Figure 4:**
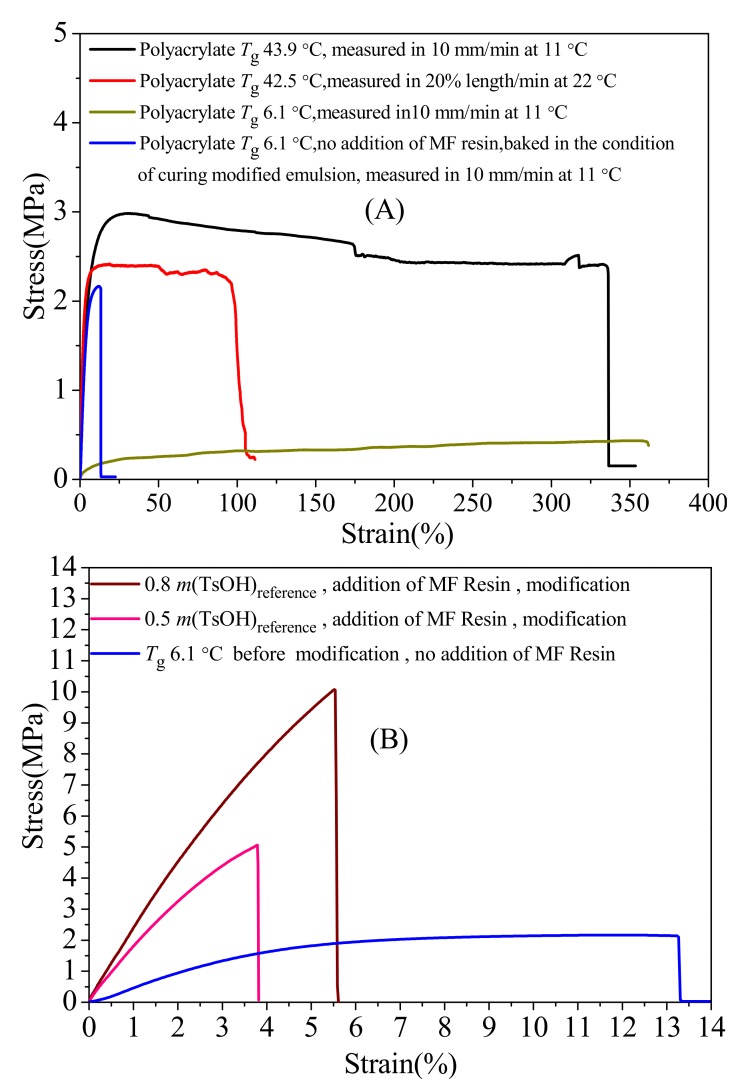
(**A**)Stress–strain curves of emulsion film (not modified by reaction polyacrylate with melamine–formaldehyde (MF) resin); and (**B**) the film baked in the condition of cured modified emulsion.

The tensile strength and the elongation at break of films were determined from the stress tensile curves shown in Figure 4. In Figure 4A, the emulsion film with *T*_g_ 43.9 °C owed the maximum tensile strength. When *T*_g_ was increased from 6.1 to 42.5 °C, the maximum tensile strength was increased. For the polymer with a *T*_g_ of 6.1 °C, the film was soft; however, it was stiffer for polymer with a *T*_g_ of 42.5 or 43.9 °C. The elongation at break of three films baked in Infrared lamp of 110 V voltage was higher than 90%. The above results manifest that higher *T*_g_ leads to higher maximum tensile strength in the same test condition. For polyacrylate with *T*_g_ 43.9 °C, polymer chains become locked [58], and chain segment motion is hard to conduct in the temperature 11 °C that is lower than *T*_g_. Therefore, higher energy is needed to be absorbed to overcome the rotational energy barriers in the polymer chain to enable the segments of the polymer chains to move [59,60] in a temperature lower than *T*_g_, leading to the increase of maximum tensile strength.

When the *T*_g_ of the emulsion film was 6.1 °C, the maximum tensile strength of the film baked under the Infrared lamp in Figure 4A was lower than that of the baked in the conditions of modified polymer (heating in 80 °C for 120 min, and then in 150 °C for 40 min). The reason may be that carboxyl of the polymer produced by dissociation of the ammonium salt can react with the hydroxyl of the HEMA units in 150 °C, and the crosslinking reaction happens. However, the elongation at break of the former was larger than that of the later. The above difference indicates that heating condition style could influence the tensile strength and the elongation at break of films.

As shown in Figure 4B, the maximum tensile strength of the film modified by reaction of polyacrylate with MF resin was higher than that of the film not modified, but the elongation at break of the former was lower than the latter. The hydroxyl of the HEMA units from the polymer chain can react with MF resin [61], and polymer with crosslinked structure can be synthesized under the catalyzation of TsOH. Polymer chain segment in crosslinked polymer is harder to motion for the strict of chemical bonding between polymer chains than that in linear polymer. In order to enable the segments of the polymer chains to move, more energy is needed to be absorbed to overcome the rotational energy barriers in crosslinked polymer than that in linear polymer, leading to the increase of maximum tensile strength. When the amount of TsOH changed from 0.5 to 0.8 *m*(TsOH)_reference_, the maximum tensile strength was increased, but the film of these two kinds was no more brittle than the film with 1.0 *m*(TsOH)_reference_. The slope of the stress–strain curves in Figure 4B indicates that the greater the amount of the catalyst TsOH, the stiffer the film is. Above all, the crosslinking reaction can reinforce the emulsion film with HEMA units in polymer chain.

## 4. Conclusions

RITP Polymerization of four kinds of acrylate were developed to synthesize emulsion latex in this study. With the addition of ammonium salt of MAA, stable white latex was obtained, indicating iodine is consumed in the polymerization, and ammonium salt of MAA can act as polymeric emulsifier in the reaction system. The stable white latex and low molecular weight distribution index (lower than 1.85) manifests that no additional control agent is needed to regulate the polymerization. The random copolymerization with high solid content (can be over 45 wt.%) was conducted in 210 min and monomer conversion (over 95%) was high. The viscosity of emulsion with HEMA was larger than that without HEMA, and the former polymers can react with MF resin to obtain cured film with properties of hardness, adhesion, and water resistance. From *T*_10%_ and *T*_50%_, the thermal stability of the polymer with HEMA was better than that without HEMA. When the MAA amount ranged from 0.7 to 1.2 *m*(MAA)_0_, the emulsion was stable in the reaction time, and minimum viscosity occurred in 1.0 *m*(MAA)_0_. However, the emulsion was not stable in 0.5 *m*(MAA)_0_, indicating that a moderate amount of ionized MAA was needed for participation in the stabilization of the emulsion. With the increasing of mass ratio of soft/hard monomer, the *T*_g_ of the emulsion film was decreased; the best results in terms of *M*_n,GPC_, *Ɖ*, *d*_p_, low viscosity, pencil hardness, adhesive property, water resistance, and better thermal stability were obtained when the mass ratio BA/MAA was 1/2. When the amount of I_2_ was increased from 0.7 to 1.1 m(I_2_)_0_, *M*_n,GPC_ was decreased from 50,100 to 35,100 g·mol^−1^, showing that emulsion *M*_n_ of emulsion can be tuned by the amount of I_2_. By Iterative one-pot emulsion copolymerization, the *M*_n,GPC_ was increased and *Ɖ* was decreased, indicating the living of the polymer. Above all, the cured film obtained by modified polymer with HEMA in the series of changing the amount of MAA, the series of changing soft/hard monomer mass, and the series of chain extension polymer in Iterative one-pot emulsion copolymerization displayed hardness, water resistance, and adhesive property, signifying that the emulsion can be potentially applied in the baking coating of metal surfaces. Furthermore, higher *T*_g_ resulted in higher value of the maximum tensile of the polymer, and the maximum tensile of the modified film was higher than that not modified. Compared with conventional emulsion polymerization, molecular weight could be controlled, and *M*_n_ of polymer synthesized in RITP emulsion polymerization is higher; emulsion of polyacrylate containing hydroxyl group monomer units prepared by RITP emulsifier-free radical polymerization is more stable. In summary, the work in this paper may provide a synthetic method of emulsion by RITP polymerization for potential industrial baking coating.

## Figures and Tables

**Table 1:** Exploratory experiment for stable emulsion by emulsifier-free reverse iodine transfer polymerization (RITP).

[*m*(BA)+*m*(BMA)]:m(MMA)	*m*(BMA): m(MMA)	*m*(BA): m(MMA)	*m*(Ammonia Solution)/g	Reaction Temperature in Reaction Time	Reaction Phenomena
1/3	1/3	0	2.045	80 °C for 120 min, then 75 °C for 60 min	milky white, high viscosity, a little coagulation
2/1	2/1	0	2.257	80 °C for 120 min, then 75 °C for 60 min	milky white, high viscosity, a little coagulation
3/1	3/1	0	2.016	80 °C for 120 min, then 75 °C for 60 min	milky white with weak blue, appropriate viscosity
4/1	4/1	0	1.991	80 °C for 120 min, then 75 °C for 60 min	milky white with weak blue, appropriate viscosity
7/2	7/2	0	1.999	80 °C for 120 min, then 75 °C for 90 min	milky white, high viscosity
7/2	3/1	1/2	1.942	80 °C for 210 min	milky white with weak blue, appropriate viscosity
7/2	3/1	1/2	0.204 a	80 °C for 220 min	milky white, a lot of coagulation

^a^ 0.204: HEMA was not added. Conditions: In this table, n(MAA)/n(ACPA)/n(I_2_) = 18/1.15/1, and the total mass of ingredient except for HEMA kept 27.462 g in theory; when HEMA (22.55 mmol) was added, the monomer mass content was 40 wt.%, in theory.

**Table 2:** Polymer synthesized by emulsifier-free RITP polymerization of acrylate.

[*m*(BMA)+*m*(BA)]:*m*(MMA)	*m*(HEMA)/g	*M*_n,th_^a^ (10^3^ g·mol^−1^)	*M*_n,GPC_ (10^4^ g·mol^−1^)	*Ɖ*	*d*_p_(nm)^b^/PDI	Viscosity (mPa·s)/Rotar Type/Rotar Rate (rpm)	Solid content (wt.%)
3/1	0	6.14	3.82	1.48	386/0.195	40/E/750	39.7
7/2	0	6.16	3.54	1.57	400/0.128	50/E/750	39.1
4/1	0	6.16	4.17	1.42	398/0.187	40/E/750	40.0
3/1	2.618	7.38	2.96	1.66	412/0.192	462/F/750	44.1
7/2	2.618	7.38	3.02	1.62	357/0.066	525/F/750	45.9
4/1	2.618	7.38	3.41	1.51	525/0.107	1170/G/750	44.0

^a^*M*_n,th_ = (mass of monomer) × (monomer conversion)/(2 × *n*_I2,initial_) + *M*_AI_, in which *M*_AI_ = 275.02 g ·mol^−1^. ^b^ d_p_: particle diameter; PDI: Polydispersity Index of Particle (PDI). Conditions: In this table, n(MAA)/n(ACPA)/n(I_2_) = 18/1.15/1, and *m*(BA)/*m*(MMA) = 1/2; 1.39 g ammonia solution (20.43 mmol-22.84 mmol NH_3_); the total mass of ingredient that HEMA is not included, and it was kept at 27.462 g, in theory.

**Table 3:** Thermal properties of the copolymer.

[*m*(BMA)+*m*(BA)]:*m*(MMA)	m(BA):m(BMA)	m(HEMA)/g	*T*_5%_(°C)	*T*_10%_(°C)	*T*_50%_(°C)	*T*_g,expermental_(°C)
3/1	1/5	0	182	253	382	-
7/2	1/6	0	137	207	382	-
4/1	1/7	0	110	207	380	-
3/1	1/5	2.618	158	237	391	30.8
7/2	1/6	2.618	167	242	391	28.0
4/1	1/7	2.618	198	256	389	36.3

Conditions: In this table n(MAA)/n(ACPA)/n(I_2_) = 18/1.15/1, and *m*(BA)/*m*(MMA) = 1/2; the total mass of ingredient that HEMA is not included, and it was kept at 27.462 g, in theory.

**Table 4:** Properties of the cured film.

[m(BMA)+m(BA)]:m(MMA)	m(BA):m(BMA)	m(HEMA)/g	Pencil Hardness	Adhesion by Scratch Experiment	Water Resistance
3/1	1/5	1.664	3H	3	Translucent
7/2	1/6	1.664	2H	0	Translucent
4/1	1/7	1.664	3H	0	Translucent

Conditions: In this table, n(HEMA)/n(MAA)/n(ACPA)/n(I_2_) = 21/18/1.15/1, and *m*(BA)/*m*(MMA) = 1/2; the total mass of ingredient was kept at 30.08 g, in theory.

**Table 5:** Effect of MAA on copolymerization.

*m*(MAA)	*M*_n,th_ (10^3^ g·mol^−1^)	*M*_n,GPC_ (10^4^ g·mol^−1^)	*Đ*	*d*_p_/PDI	Viscosity (mPa.s)/Rotar Type/Rotar Rate (rpm)	Emulsion Appearance
0.5 m(MAA)_0_^a^	-	-	-	-	-	Large pieces of flocculation
0.7 m(MAA)_0_	6.90	3.27	1.53	364/0.147	1320/F/75	Milky white with blue
m(MAA)_0_	7.22	3.22	1.56	409/0.49	1460F/75	Milky white with blue
1.0 m(MAA)_0_	7.38	3.02	1.62	357/0.066	197/E/75	Milky white with Weak blue
1.1 m(MAA)_0_	7.53	2.61	1.76	501/0.138	1800/F/75	Milky white
1.2 m(MAA)_0_	7.69	2.93	1.65	438/0.185	1580/F/75	Milky white with Weak blue

^a^*m*(MAA)_0_ = 1.664 g. Conditions: *m*(BA)/*m*(MMA)/m(BMA) = 1/2/6; except for the changing amount of MAA and corresponding ammonia solution used for neutralization, the total mass of the ingredient was kept at 30.08 g, in theory; n(HEMA)/n(BMA)/n(BA)/n(MMA)/n(ACPA)/n(I_2_) = 21/40.36/7.46/19.1/1.15/1.

**Table 6:** Effect of MAA on thermal stability of the copolymer.

Run	*m*(MAA)	*T*_5%_ (°C)	*T*_10%_ (°C)	*T*_50%_ (°C)	*T*_max_ (°C)
1	0.7 *m*(MAA) _0_	203	266	389	388
2	0.9 *m*(MAA)_0_	205	272	388	387
3	1.0 *m*(MAA) _0_	167	242	391	390
4	1.1 *m*(MAA) _0_	141	229	387	391
5	1.2 *m*(MAA) _0_	166	242	391	390

**Table 7:** Effect of MAA on properties of the cured film.

Run	*m*(MAA)	Pencil Hardness	Adhesion Judged by Scratch Experiment	Water Resistance
1	0.7 m(MAA) 0	2H	0	Translucent
2	0.9 m(MAA) 0	2H	0	Translucent
3	1.0 m(MAA) 0	2H	0	Translucent
4	1.1 m(MAA)0	2H	1	Translucent
5	1.2 m(MAA) 0	2H	1	Whitening

**Table 8:** Effect of soft/hard monomer mass ratio on copolymerization.

m(BA):m(MMA)	Conversion (%)	*M*_n,th_ (10^3^ g·mol^−1^)	*M*_n,GPC_ (10^4^ g·mol^−1^)	*Ɖ*	Viscosity (mPa.s)/Rotar Type/Rotar Rate (rpm)
1/4	>99.5	7.38	2.80	1.70	2020/F/75
1/3	>99.5	7.38	2.49	1.82	1650/F/75
1/2	>99.5	7.38	3.02	1.62	197/E/75
1/1	97.2	7.18	2.71	1.74	2520/F/75
4/3	>99.5	7.38	2.91	1.64	258/E/75
3/2	>99.5	7.38	2.60	1.77	549/E/75

Conditions: n(HEMA)/n(MAA)/n(MMA)/n(ACPA)/n(I_2_) = 21/18/19.1/1.15/1, and [m(BMA)+m(BA)]:m(MMA) = 7/2; 1.39 g ammonia solution (20.43–22.84 mmol NH_3_); the total mass of the ingredient was kept at 30.08 g, in theory, and total solids’ content remained fixed.

**Table 9:** Effect of soft/hard monomer mass ratio on thermal properties of the copolymer.

m(BA):m(MMA)	*T*_5%_ (°C)	*T*_10%_ (°C)	*T*_50%_ (°C)	*T*_max_ (°C)	*T*_g,expermental_ (°C)
1/4	156	242	388	387	43.9
1/3	153	237	391	392	42.7
1/2	167	242	391	390	28.0
1/1	139	207	388	389	22.2
4/3	175	248	388	388	14.2
3/2	192	256	386	387	6.1

**Table 10:** Effect of soft/hard monomer mass ratio on properties of the cured film.

m(BA):m(MMA)	Pencil Hardness	Adhesion Judged by Scratch Experiment	Water Resistance
1/4	3H	3	Whitening
1/3	2H	0	Whitening
1/2	2H	0	Translucent
1/1	2H	0	Translucent
4/3	2H	1	Whitening
3/2	2H	4	Whitening

**Table 11:** Effect of I_2_ on copolymerization.

*m*(I_2_):*m*(I_2_)_0_^a^	Time(min)	Conversion (%)	*M*_n,th_ (10^3^ g·mol^−1^)	*M*_n,GPC_ (10^4^ g·mol^−1^)	*Đ*	d_p_/PDI	Solid Content (wt.%)
0.7 m(I2)0	210	>99.5	8.68	5.01	1.26	394/0.040	36.9
0.8 m(I2) 0	210	>99.5	7.63	4.96	1.24	381/0.015	37.2
0.9 m(I2) 0	210	>99.5	6.81	4.42	1.33	386/0.034	37.1
1.0 m(I2)0	214	>99.5	6.16	3.54	1.57	400/0.128	39.1
1.1 m(I2) 0	210	98.5	5.62	3.51	1.50	333/0.035	36.6

^a^*m*(I_2_)_0_ = 273 mg. Conditions: n(I_2_)_0_ = 1.074 mmol; n(MAA)/n(BMA)/n(BA)/n(MMA)/n(ACPA)/n(I_2_)_0_ = 18/40.36/7.46/19.1/1.15/1; *m*(BA)/*m*(MMA)/m(BMA) = 1/2/6; 1.39 g ammonia solution (20.43 mmol–22.84 mmol NH_3_); the total mass of the ingredient kept 30.08 g in theory.

**Table 12:** Iterative one-pot emulsion copolymerization.

Type	Time (min)	*M*_n,th_ (10^3^ g·mol^−1^)	*M*_n,GPC_ (10^4^g·mol^−1^)	*Đ*	*d*_p_/PDI	Solid Content (wt.%)
Poly(MAAa-co-HEMA-co-BMA) latex	170	3.52	2.54	1.60	283/0.013	36.3
Block copolymer Poly(MAA-co-HEMA-co-BMA)-b-poly(BA-co-MMA)	90	4.98	3.24	1.55	323/0.028	45.8
Poly(MAA-co-BMA) latex	140	2.71	2.55	1.77	243/0.048	27.9
Block copolymer Poly(MAA-co-BMA)-b-poly(BA-co-MMA)	90	4.20	2.94	1.68	414/0.145	40.5

^a^ MAA in this table was ionized by neutralization with ammonia solution.

**Table 13:** Thermal stability of the copolymer in Iterative one-pot emulsion copolymerization.

Type	*T*_5%_ (°C)	*T*_10%_ (°C)	*T*_50%_ (°C)	*T*_max_ (°C)
Poly(MAA^a^-co-HEMA-co-BMA)latex	115	222	395	408
Block copolymer Poly(MAA-co-HEMA-co-BMA)-b-poly(BA-co-MMA)	206	256	388	396
Poly(MAA-co-BMA) latex	120	197	389	397
Block copolymer Poly(MAA-co-BMA)-b-poly(BA-co-MMA)	184	268	380	401

^a^ MAA in this table was ionized by neutralization with ammonia solution.
